# The Implication of Physiological Ketosis on The Cognitive Brain: A Narrative Review

**DOI:** 10.3390/nu14030513

**Published:** 2022-01-25

**Authors:** Mansour Altayyar, Jennifer A. Nasser, Dimitra Thomopoulos, Michael Bruneau

**Affiliations:** 1 Department of Nutrition Sciences, Drexel University, 1601 Cherry Street, Philadelphia, PA 19102, USA; jan57@drexel.edu (J.A.N.); dnt38@drexel.edu (D.T.); 2 Department of Health Science, Drexel University, 1601 Cherry Street, Philadelphia, PA 19102, USA; mlb425@drexel.edu

**Keywords:** ketosis, cognition, ketogenic diet, intermittent fasting, medium-chain triglycerides, neuroimaging, ketone bodies

## Abstract

Optimal cognitive functions are necessary for activities of daily living and self-independence. Cognitive abilities are acquired during early childhood as part of progressive neurodevelopmental milestones; unfortunately, regressive changes can occur as part of physiological aging, or more ominously, pathological diseases, such as Alzheimer’s disease (AD). Cases of AD and its milder subset, mild cognitive impairment (MCI), are rising and would impose a burdensome impact beyond the individual level. Various dietary and nutritional approaches have potential for promising results in managing cognitive deterioration. Glucose is the core source of bioenergy in the body; however, glucose brain metabolism could be affected in aging cells or due to disease development. Ketone bodies are an efficient alternate fuel source that could compensate for the deficient glycolytic metabolism upon their supra-physiologic availability in the blood (ketosis), which, in turn, could promote cognitive benefits and tackle disease progression. In this review, we describe the potential of ketogenic approaches to produce cognitive benefits in healthy individuals, as well as those with MCI and AD. Neurophysiological changes of the cognitive brain in response to ketosis through neuroimaging modalities are also described in this review to provide insight into the ketogenic effect on the brain outside the framework of purely molecular explanations.

## 1. Introduction

Alteration of normally acquired cognitive function has a significant impact on the affected individual’s life. Moreover, the burden of cognitive deterioration in conditions such as Alzheimer’s disease (AD) and mild cognitive impairment (MCI) extend beyond the individual to include caregivers and the entire healthcare system [1]. In 2021, AD was estimated to affect around 6.25 million people living in the United States. Moreover, the projected number of AD cases is expected to steadily increase to 13.9 million by the year 2060 [2]. This ominous trend is also predicted for MCI with estimated cases expected to be double the number of AD in the United States [2]. Thus, efforts to tackle the growing number of cognition-impairing diseases and conditions are of public health importance; high-quality research efforts addressing cognitive impairment are necessary.

Functionality of the normal brain is essential for optimizing activities of daily living. The brain’s functional sophistication is not constrained to motor movements and autonomic regulations—higher neurocognitive functions needed for learning processes, social skills, and interactive situations are also functionally relevant [3,4]. Neurocognitive abilities are essential for humans to interact with their surrounding environment and acquiring these abilities is a progressive process; thus, advancement in cognitive functioning mirrors normal neurodevelopmental changes beginning in infancy and early childhood [5,6], up to older adulthood, where regressive changes (structural and functional) may occur and may impact some of the previously acquired cognitive abilities [7,8]. MCI, on the other hand, is an alarming condition that represents a transitional state from cognitive aging to a more pathological subset [9,10]. The risk of MCI increases with age [11], but unlike AD, it can be slowed with early detection and management [12]. The high prevalence of AD and MCI highlights the fact that they are conditions of major concern; however, other conditions that cause cognitive deterioration exist (e.g., Parkinson’s disease, frontotemporal degeneration, autoimmune-related disorders, Huntington disease, and vascular dementia), and some may even emerge early in life (≤65 years) or even younger (≤45 years old) [13].

The current pharmacological and non-pharmacological options to manage cognitive deterioration are not fully established and have modest outcomes [14,15]. Although drug development to manage this issue is active and ongoing [16], the FDA has not approved any new drugs to treat cognitive deterioration since 2003, except for Aducanumab which has only recently been approved. Even though Aducanumab is subject to further efficacy and safety evaluation [17,18], the FDA’s accelerated approval highlights the crucial need for cognitive disease therapies [17].

The emerging information from research employing nutrition-based interventions to manage cognitive decline is gaining acceptance [19,20]. Because of their feasibility, accessibility, and tolerability advantages, nutritional approaches can be used as an alternative to drug therapy or in conjunction with them. In fact, as detailed in the Canadian Consensus Conference on the Diagnosis and Treatment of Dementia (CCCDTD), nutrition-based intervention is one of the recommended approaches to reduce the risk of developing dementia and cognitive impairment in the Canadian clinical guidelines [21]. Relying on nutritional interventions may therefore become a preferred, non-pharmacologic lifestyle therapy in situations where traditional pharmacological remedies are contraindicated or non-applicable.

Occasionally, adherence to a special diet is bound up with an overall switch to a healthier lifestyle. Nevertheless, some diets are richer in particular nutrient(s) than others. These nutrients may contribute to neuroprotection and cognitive enhancement by triggering a series of molecular modulations that contribute to anti-neuroinflammatory and antioxidative processes [19,20], and orchestrate cellular maintenance roles (i.e., apoptosis and neurogenesis) to support the neurocognitive brain [20]. Ketone bodies have also been described as nutritional biomarkers carrying antioxidative processes that promote mitochondrial integrity and support neurosynaptic function [22,23]. Furthermore, β-hydroxybutyrate (BHB) drives an anti-inflammatory cascade that counteracts AD pathology [24].

In addition to molecular modulation, ketone bodies contribute to the brain’s bioenergy that could support cognitive abilities [25] and, because metabolic bioenergy is one of targets of drug models designed to tackle cognitive diseases [16], it is of great importance to evaluate trends in basic and clinical research relating to the role of ketone bodies in cognition. In this narrative review, we review possible benefits of ketogenic diets on cognitive function for those living with MCI and AD and present some neurophysiological outcomes germane to this issue.

## 2. The Ketogenic Diet Effect on Neurocognition

### 2.1. Ketogenic Diet Ketosis: Biological Influences

Acetoacetate, BHB, and acetone are compounds collectively known as ketone bodies (or ketones); they are products of serial biochemical reactions related to fatty acid oxidation. Unlike acetone, which is a nonmetabolizable byproduct, BHB, and acetoacetate are channeled to the peripheral tissues for metabolism and energy. The brain also uses these ketones to meet its energy demands, especially under situations of glucose scarcity, which generally require ketones to be the body’s alternative fuel [26,27]. The elevation of ketones in the blood, generally known as ketosis, can be intentionally and nutritionally induced to raise physiological levels via several methodologies that partially mimic starvation and carbohydrate depletion [26,28].

Ketogenic diets (KD), which rely on low-carbohydrate and high-fat intake, were first described by Wilder [29] to treat epilepsy in children. The diet was also listed among dietary approaches that have been previously demonstrated to aid cognitive impairment prevention and improve cognition [19]. The literature contains several animal and human studies that confirm the favorable contribution of KD to multiple neurological illnesses, including AD and related dementias. Physiological ketosis achieved by KD is believed to be the core process responsible for favorable neurological outcomes [26]. At the molecular level, ketosis can produce an efficient antioxidative effect by enhancing mitochondrial biogenesis and reducing reactive oxygen species through numerous modulations of intracellular signaling factors, such as the nuclear factor erythroid 2-related factor 2 (Nrf2) and AMPK/mTOR pathways. Ketosis can also drive anti-inflammatory processes, promote key synaptic transmission modulations, and protect against amyloid and tau protein deposition [30,31,32]. These molecular-level explanations are of great value for understanding how the body adapts to ketosis and subsequently impacts overall health. They aid in expanding on existing ketogenic research models research and pave the way for clinical applications.

### 2.2. Ketogenic Diet Molecular Adaptation

In an animal investigation, young and aged rats assigned to KD (75.85% fat and 3.85% carbohydrates) had sustained ketosis beginning from the first week of the intervention and throughout the entire experiment period (12 weeks), unlike rats assigned to a control diet (16.35% fat and 64.89% carbohydrates). Interestingly, the authors demonstrated improved performance in a working memory bi-conditional association task that reflect prefrontal cortex (PFC) and hippocampal interaction [33]. Biochemical and molecular changes have also been reported; animals on KD had higher expression of monocarboxylate transporter 1 (MCT1) and MCT4 at the PFC [33], which are fundamental transporters that allow for cellular and neuronal entry of ketones [34]. The expression of VGAT, a GABA transporter, was also increased at the PFC similar to the hippocampus [33]. However, there was a decrease in the glucose transporter (GLUT1) at the PFC. These findings not only provide evidence of the working memory advantages of ketosis but also the biochemical adaptations occurring within certain brain areas, most notably the molecular resilience to switch between energy substrate metabolism expression-related transporters depending on peripheral availability. Medium-chain triglycerides (MCT) were supplemented to the KD protocol, which could have produced an additive effect, perhaps unobserved or not achieved with KD alone.

### 2.3. Ketogenic Diet Ketosis and Human Cognition

Krikorian, Shidler [35] have described the beneficial effect of KD on cognition and its possible capacity to deter MCI progression. In their study, older participants with MCI were assigned to either a high-carbohydrate diet (50% of calories) or a low-carbohydrate diet (5–10% of calories) for six weeks; those assigned to the low-carbohydrate diet consumed only an equivalent of 20 g of carbohydrates per day in order to trigger and sustain ketosis. The findings indicated that the low-carbohydrate diet group had notably lower caloric intake in general which was primarily attributed to the lower carbohydrate consumption. However, the same group experienced notably higher fat and protein intakes (a diet typically representing KD). This particular group had improved secondary memory and verbal paired-associate learning task performance. There was an unspecified difference between the groups regarding the Trail Making B test, which was intended to test cognitive working memory [35]. At the same time, urinary ketones were only detected for the low-carbohydrate group with a mean increase of 5.4 mg/dL [35]. The researchers found that ketone concentrations were significantly correlated to memory performance (*r* = 0.45). Although a reduction in insulin could be a contributor to the cognitive findings (*r* = 0.47), the authors did not report correlational statistical significance (*p* = 0.11). The other improved anthropometric and biochemical factors did not relate to improvements in cognitive performance [35]. For this reason, adhering to KD might not only lead to cognitive improvements, but may do so within a short period, which has critical implications for the progression of MCI to AD.

A large, year-long RCT investigated the effect of two different calorie-restrictive diets on cognition and mood in overweight and obese individuals [36]. One arm of the dietary interventions was assigned a low-fat diet (46% carbohydrate and 30% total fat; <8% saturated) and the other arm involved a low-carbohydrate diet composed of 20–40 g of carbohydrates (4% of energy) and a higher amount of fats (61% of energy, 20% saturated). The composition of the latter diet is representative of KD. In addition to weight loss following KD, working memory cognitive function measured with the Digit Span Backward test improved from baseline, which provides clues as to the additional long-term benefits of KD beyond weight loss [36]. However, these findings were matchable to the contrasting intervention [36]. Plasma glucose and serum insulin also were measurably reduced in both interventions [36]. Based on these findings, evidence of macronutrient contribution to cognitive improvements was not established; however, assuming that the KD diet did lead to ketosis, it is possible that the cognitive improvement occurred through different mechanisms attributed to each diet, or perhaps a common molecular adaptation shared between both diets with a relationship to weight loss, glycemic improvement, or others (e.g., anti-inflammatory processes).

### 2.4. Ketogenic Diet and Cognition: The Challenge of the ApoE4 Gene

As mentioned previously, various researchers have suggested that ketogenic dietary interventions can indeed slow down functional cognitive decline and the development of cognition-impairing diseases; however, the benefit of ketosis produced by KD could be limited to those without the apolipoprotein E4 (ApoE4) variant [30,31,32], a variant known for its association with AD [37]. However, a case study of a heterozygous ApoE4 morbidly obese 71-year-old woman with metabolic syndrome and mild MCI/AD with a positive family history of progressive cognitive impairment showed a significant improvement in memory-related cognitive functioning measured by the Montreal Cognitive Assessment (MoCA) after 10 weeks of KD. The intervention was intended to sustain plasma ketones between 0.5 and 2.0 mg/dL together with physical and mental exercises [38]. Similarly, an obese 68-year-old male with heterozygous ApoE4 mild AD and diabetes mellitus type 2 showed improvement on the MoCA scale representing AD regression from 23/30 to 29/30 after 10 weeks of KD. In the latter case, a hybrid KD approach was applied with time-restricted intermittent fasting (IF) applied three days per week [39]. In both cases, improvements in several metabolic parameters (e.g., glucose, glycosylated hemoglobin, insulin, and lipid profile) were documented [38,39]. Although there were multiple elements that could have contributed to the observed cognitive improvements, these case studies have provided breakthrough evidence of the potential to slow down or reverse MCI from progressing into AD through ketogenic dietary interventions, even in ApoE4+ cases.

## 3. Medium Chain Triglycerides (MCT) Ketosis and Cognitive Advantages

### 3.1. MCT Ketosis

Adherence to strict dietary patterns could be challenging; for example, a study on young and middle-aged individuals with epilepsy who were asked to adhere to KD to manage seizures suffered from a large number of dropouts. However, those who adhered to the diet had promising outcomes [40]. It is therefore sensible to examine different methods by which ketosis can be induced and adhered to. This may be particularly beneficial for those who may experience more challenges in adhering to strict diets (e.g., elderly adults, demented adults, or children). Since the ingestion of MCT can promote the rapid production of ketone bodies [41], MCT products may be considered a feasible option for some cohorts. A single dose of 20 mL of MCT containing caprylic acid (C8) can promote ketosis within minutes and sustains it for up to 4 h [41].

The MCT structure is made up of fatty acids that are composed of carbon chain lengths between 6 and 12 (medium-chain fatty acids, MCFA). Unlike long-chain fatty acids (LCFA), which are composed of a more than 12-carbon chains, MCFA does not require incorporation into micelles and are readily absorbed through the intestinal lumen. They are then bound to albumin and are channeled to the liver via the portal circulation [42]. When the liver is packed with fatty acids and their β-oxidation product (i.e., acetyl CoA), ketogenesis occurs [26,27]. The ketones are released into the circulation thereafter and enter the peripheral tissues for ketolysis and utilization [26,27].

### 3.2. MCT Intake: Ketosis and Cognitive Outcomes in AD and MCI

The cognitive benefits of MCT supplementation have been noted in multiple research studies (Table 1). Improvements in multiple cognitive domains, including episodic memory and executive functioning, were noted after six months of bidaily 15 g intake of MCT in 39 participants with MCI compared to 44 participants on an equivalent, non-ketogenic placebo. A marked elevation in plasma ketones was noted only in those assigned to MCT, and this elevation was directly and significantly correlated to cognitive improvements [43].

Similar findings were presented in an earlier 6-months trial [45], in which participants with MCI were assigned to two different arms, a daily 56 g of MCT oil arm or a placebo (canola oil). The study participants assigned to the placebo had no changes in BHB or cognitive performance. However, in the MCT arm, a participant who lacked the ApoE4 gene had an increase in BHB compared to baseline, which declined later in subsequent weeks. This was different from the other participant who possessed the ApoE gene and sustained an increase in BHB throughout the entire study period. Concerning cognition, both participants showed improvements in their cognition, as measured by the Alzheimer’s Disease Assessment Cognitive Subscale (ADAS-Cog), but the improvement was more prominent in the ApoE4 negative individual [45]. In line with this finding, an earlier study demonstrated that a single 40 mL dose of MCT raised ketone bodies steadily over two hours in participants with amnestic MCI and probable AD. Although all participants had cognitive impairments upon initial evaluation, improvements were only noted in ApoE4− participants post-intervention [50]. Despite ketosis, ApoE4+ participants failed to experience enhancements in cognition [50]. Notably, ApoE4+ participants sustained a continuous increment in ketone bodies, unlike ApoE4− individuals [50], which is somewhat comparable to the previously described study conducted by Rebello et al [45].

A feasibility and efficacy study on individuals with very mild, mild, and moderate AD showed cognitive improvement on the Mini-Mental State Examination Scale (MMSE) and ADAS-Cog after three months of MCT-supplemented KD [44]. However, the improvement in cognitive function did not persist, and eventually reverted to baseline after only one month of dietary cessation. Notably, ketone body measurements changed concurrently; their measurements indicated return to baseline after the dietary intervention concluded [44]. These findings indicate the efficacy of ketosis on the cognitive brain and the importance of its long-term maintenance once it is achieved. Moreover, this was demonstrated again in another study [48], although cognitive enhancements were not observed instantly in AD patients [48] compared to undemented counterparts using 20 g of MCT meal [49], prolonged intervention for three months did eventually lead to improvements in working memory, short-term memory, and processing speed in people with AD [48]. This finding stresses a point that we have alluded to earlier about the requirement for prolonged ketonic adaptation in some groups to acquire cognitive benefits.

Additional evidence of MCT supplementation benefits in those with mild to moderate AD was shown in a recent randomized crossover placebo-controlled trial which indicated that the consumption of 17.3 g of MCT jellies throughout 30 days with meals led to marked acetoacetate and BHB elevation in addition to improvements in multiple ADAS-Cog scales, including those which are particularly memory-related [46]. Contrary to the MCT intervention, the placebo (canola oil) was associated with worsened ADAS-Cog scores, highlighting the desirable ketogenic effects on cognitive abilities in those with MCI and AD. Nevertheless, other factors in the results should be considered, one of which is changes in lipid metabolism, including oleic acid, palmitic acid, linoleic acid, and lysophosphatidylcholines, that may have contributed to the cognitive outcomes. Additionally, the researchers indicated that improvements in cognitive function presented by those who were ApoE4−, while ApoE4+ had no cognitive benefits from the intervention, but it is worth noting that ApoE4+ participants accounted for only 6% of the study sample.

### 3.3. MCT Ketosis and the ApoE4 Gene

In addition to Xu et al [46] and Rebello et al [45], the resistance of cognitive improvements via MCT ketosis was reported in earlier studies [50,51]. Whether a single-dose of 40 mL of MCT was applied [50] or three months adherence to a daily 20 g of 95% caprylic acid (C8) MCT [51], cognitive benefits in those with mild to moderate AD were absent in ApoE4 carriers compared to ApoE4− despite the marked rise in ketone bodies achieved by both interventions. This finding was in contrast to the findings found by Fortier et al [43], who reported cognitive enhancements with MCT regardless of ApoE4 status, and dissimilar to a case study portraying cognitive benefit from KD in an ApoE4+ individual [38]. Moreover, the few ApoE4+ samples from studies conducted to date render these findings inconclusive for this sub-group [45,46,50]. It is possible that more prolonged adaptation or higher doses are required in ApoE4 carriers to overcome cognitive deterioration; that being said, the concept of bioenergy-driven cognitive enhancement may not be of great value for some individuals unless concurrent adjustment to underlying molecular mechanisms occurs as well.

### 3.4. MCT Ketosis in Cognitively Intact Individuals

MCT products are widely available for users to purchase for the apparent “Keto” benefits obtained for weight loss; however, the question of whether these MCT products can enhance cognitive capacity in people with and without cognitive deficits remains questionable. In a double-blind placebo-controlled trial, Ota et al. (2016) suggested that acute ketosis induction can lead to swift cognitive improvements in older adults free from dementia [49]. In this study, participants ingested a single meal containing 20 g of MCT, facilitating a gradual increase in plasma acetoacetate and BHB within the first post-ingestion hour that was sustained beyond three hours [49]. These changes in ketogenic biomarkers were met with improvements in some cognitive tests compared to equivalent meals without MCT, especially among those with relatively lower initial global cognitive scores [49].

The instant favorable outcomes of MCT consumption on cognitive abilities in older people free from dementia warrant further exploration in research to understand the mechanisms for how acute changes (metabolic and physiologic) affect the brain upon swift ketone body increments, especially because these findings were not replicated in those with mild to moderate AD [48]. Furthermore, MCT-induced ketosis may lead to cognitive enhancements in individuals outside the frame of advanced age. A recent study by Ashton et al [47] in younger healthy adults has revealed that MCT supplementation over four weeks produced gradual improvements in some cognitive tests. Compared to a carbohydrate placebo, daily consumption of either 12 g or 18 g of MCT significantly improved cognition in multiple domains, including the memory-related domain [47]. The authors postulated possible ketogenic effects but did not measure ketones. This study demonstrated the favorable effect of MCT on cognitive function in young adults, but the biochemical correlations highlighted within our review remain obscure and warrant further investigation.

### 3.5. MCT Interventions and Future Research

Although trends for data with MCT and cognition seem promising, more research is necessary. First, the current data rely on relatively few participants. Second, MCT composition and dose is vastly variable between studies with C8 and capric acid (C10) doses being between 30% and 70%, respectively, in the Ashton et al study [47] and 60:40 (C8:C10) in the Fortier et al study [43]; the latter could be more ketogenic because of its higher C8 concentration [41]. Hence, studies need to accumulate to find a consensus on which MCT doses and composition could achieve cognitive benefits. Third, findings from ApoE4+ carriers are inconclusive, and requirements for unique MCT interventions with higher doses, more extended periods, or both, may be warranted for accurate examination in this subgroup. Finally, use of MCT to induce ketosis remains limited by feasibility issues, especially for those who may benefit from ketosis (e.g., older people and AD patients). For instance, all the participants who were classified as moderate AD in the Taylor et al study have dropped out due to caregiver burden, and the results only represented those with very mild and mild AD [44]. Furthermore, because MCT is associated with side effects [25], long term supplementation could be challenging [43,45,51] but necessary to conserve cognitive function [44,51], further supporting the premise that research must identify novel strategies to improve compliance with approaches permitting spontaneous integration of MCT supplements to ease their inherent and common side effects.

## 4. Intermittent Fasting (IF): Ketogenic and Cognitive Potentials

### 4.1. IF Metabolic Fingerprinting

IF is a programmed time-dependent caloric restriction strategy currently trending as a weight-reduction method [52]. Different from common calorie restriction modalities requiring careful calorie counting, IF programs rely on time-counting with less rigor over caloric intake. People have been practicing fasting for thousands of years, mostly for religious reasons. Nowadays, the popularity of IF is growing beyond the scope of religion because of its portrayed feasibility and increased media dissemination. Book guides, such as *The Fast Diet—Revised & Updated: Lose Weight, Stay Healthy* [53] and *The Every-Other-Day Diet: The Diet That Lets You Eat All You Want (Half the Time) and Keep the Weight Off, and Live Longer with the Simple Secret of Intermittent Fasting* [54], in addition to a variety of smartphone applications, are currently available to help users adhere to IF. Accordingly, a plethora of research has focused on investigating IF’s clinical implications beyond weight loss (e.g., cardiometabolic benefits).

There are multiple forms of IF, including alternate-day fasting, whole-day fasting, and time-restricted fasting. Under each, there are subsets that apply specific modifications (e.g., early time-restricted fasting vs. late time-restricted fasting), but in general, they all share two common components: fasting and feeding windows. Most of the IF protocols are considered to be within the frame of short-term fasting [55,56], which means that the diet falls into a postabsorptive state instead of starvation, which makes it biochemically and physiologically unique [56]. Adherence to IF can indeed lead to substantial weight loss [57,58,59]; a three week alternate-fasting regimen was found to contribute to 0.5% weight loss in people free from obesity [60] and reduce BMI by more than 1 kg/m^2^ within four weeks [59]. People living with overweight and obesity showed the same trend [58]. Yet, the sum of weight loss is similar to other traditional caloric restriction strategies. However, changes in body composition and tissue mass are unequal [59,61,62,63] with a notable tendency for lean mass loss with IF [58,60,61,64].

Regardless of weight loss due to the rhythmic alteration in feeding and fasting windows in IF protocols, corresponding metabolic and physiologic characteristics are anticipated. Some researchers were able to report improvements in glucose, lipids, insulin, and other measures with IF [55,57,59,61,65], but others failed to demonstrate similar metabolic consequences [57,61,64]. The Tinsley and La Bounty review reported that alternate-day fasting may reduce total cholesterol between 10–21% and triglycerides by 42% in people with BMIs categorized as within the recommended range [55]. A more recent review compared alternate-day fasting with time-restricted fasting claimed that both IF methods can lead to weight loss and improve some metabolic factors known to be associated with obesity, including glucose, insulin, and lipid metabolism [61]. Furthermore, time-restricted IF may decrease hunger and increase satiety [61], although the studies contained within this review were confined to studies published in 2018 and after, making the findings meaningful but not fully conclusive.

Alterations in metabolic biomarkers are predicted in IF due to the presence of circadian changes and caloric deficits [58,65]. In a matter of four days, early form time-restricted IF (fasting between 2 p.m. and 8 a.m.) was found to increase total cholesterol in the morning through combined elevation in both LDL and HDL without marked changes in triglycerides [65], indicating that changes in triglycerides may therefore require longer interventions [58]. A feasibility study conducted by Chow et al. demonstrated that a time-restricted eating regimen with approximately 8 h of unrestricted eating can reduce eating occasions, and consequently weight, in non-diabetic adults with overweight and obesity [58]. Although there were no changes in metabolic parameters between those who adhered to time-restricted fasting vs. control (*ad libitum* eating), significant reductions in fasting glucose (−7.7%) and triglycerides (−23.6%) were noted in the IF group pre vs. post-intervention (12 weeks). No changes were observed in HbA1C nor insulin sensitivity, possibly due to the initial non-diabetic characteristics of the study participants [58]. Using almost a similar interventional approach and period with a larger sample size (*n* = 116), Lowe et al found no significant metabolic changes within the group who adhered to IF or control [64]. Only a modest decrease in weight and diastolic blood pressure was detected after IF; however, the change was not statistically significant compared to the control [64]. Of note, compliance for this study was self-reported, which may question the absolute findings unlike the Chow et al study which addressed guided mobile applications [58]. 

A short-term alternate-day fasting protocol may lead to reductions in triglycerides in men, while women may have increases in HDL [60]. Gender differences in metabolic parameters were not detected in a longer-term alternate-day fasting regimen [63]; in contrast, only initial elevation of HDL in the first six months followed by an increase in LDL in the subsequent six months was reported, with no concurrent changes in glucose, triglycerides, or C-reactive protein [63]. Nevertheless, it is thought that IF could potentially impact physiological or disease-related outcomes related to the cardiovascular system, insulin sensitivity, aging, and cognition [56,66]. This may have taken place via triggering of various distinguishable cellular and metabolic adaptations, including glucose metabolism, insulin sensitivity, lipid and, ketone metabolism [56,61,65], and modulation of inflammatory and oxidative processes [56,57,66]. Hence, there is an opportunity to conduct more focused research about IF to assess its additive effects beyond the boundary of weight reduction as a means of examining whether IF has health-related potential for facilitation and application in clinical settings. Consequently, such studies would broaden the implications of IF for non-weight loss reasons, as seen in KD.

### 4.2. Cognitive Functioning and Fasting: Implications for AD and MCI

About 100 years ago, and prior to the work of Wilder [29], fasting was the initial treatment for epilepsy. In fact, fasting was the pivotal step leading to the development of KD because of the noticeable ketonemia that impacted the disease’s outcomes [67]. From this early evidence, IF clearly manifests neurologically and may provide therapeutic benefits for those with chronic disease. The current evidence suggests that IF can contribute to the treatment of stroke, multiple sclerosis, and neurodegenerative diseases, such as Parkinson’s and AD [66,68]. The rise in ketones associated with IF also has important clinical implications that may enhance neurogenesis and neuronal cell functioning by inducing the transcription of other additional signaling factors, including brain-derived neurotrophic factor (BDNF) [22,65,66,69]. Moreover, as we highlighted previously, an elevation of blood ketones could trigger molecular signaling responsible for the efficient neuroprotective anti-inflammatory and antioxidative mechanisms responsible for maintaining mitochondrial integrity and its metabolic mechanisms [22,32].

IF’s protective role against neuronal brain damage was demonstrated previously in an animal model [70]. More recently, Park et al employed an animal model with AD to investigate how diets could improve memory and inflect beneficial outcomes for dementia [71]. Cumulatively, the investigators reported that IF for eight weeks improved memory and lowered brain beta amyloid accumulation, unlike KD, which have been shown to increase beta amyloid deposition. Although KD and IF both increased ketones, the findings were unequal with regard to neuroprotection and cognitive enhancement [71]. Notably, gut microbiota were disrupted due to KD but improved with IF [71], indicating that the gut-brain-axis interaction may be an important driver for cognitive function [68]. Park et al. indicated that an overnight IF (16–17 h fasting) could beneficially be applied in older adults as it could improve cognition [71]. Nevertheless, translation into clinical practice requires additional knowledge and exploration in both animal and human studies.

Ooi et al. recruited 99 participants with MCI and found that after a 3-year follow up, significant cognitive improvements were documented in those who regularly (*n* = 37) and irregularly (*n* = 35) practiced IF compared to non-practicing participants (*n* = 37) [72]. The metabolic assessment was performed on 15 participants from each group, and ketones (BHB and acetoacetate) were significantly detected in the blood of those who only practiced IF. Notwithstanding this, BHB notably was highest in those who practiced regular IF [72]. In addition, upon biochemical pathway analyses, ketone synthesis and degradation had the highest average impact value of 0.9 (*p* < 0.05) compared to other metabolites, which suggest active ketogenesis and ketolysis biochemistry [72]. Subsequently, 9 out of 37 of those who practiced IF regularly were placed in the successful aging category classification, while 27 reverted into normal aging and only one maintained MCI [72]. This observation readdresses the purported positive effects of IF on aging [59,65] and possible reversal of cognitive dysfunction. Although this premise could be due to the anti-inflammatory and antioxidative properties and mechanisms of action germane to IF, ketone metabolism may also play a major role [72]. Nevertheless, the author’s protocol included a unique form of Islamic fasting where a combination of time-restricted fasting (from sunrise to sunset) with weekly alternate-day fasting (Monday and Thursday) was applied that is different from Ramadan’s. Participants who practiced fasting regularly were doing so at least 12 months before the study which implies a possible long-term effect, so additional research exploring MCI and cognitive abilities prior to the study period is needed to fully evaluate the magnitude of IF on cognition.

### 4.3. Fasting and Cognition in Healthy Individuals: Outside the Frame of Weight Loss

In healthy young adults, studies exploring the effects of fasting on cognition have been largely inconclusive [73]. Harder-Lauridsen et al reported that daily fasting for 14 h over 28 days in males did not produce any remarkable advantages or disadvantages in cognitive abilities as measured by tests evaluating memory, attention, and executive function [74]. However, the cognitive tests were performed two days after fasting [74]. Iqbal et al. tested cognition after two weeks of Ramadan fasting (one month) in males and found similar cognitive outcomes but significant improvements in spatial working memory [75]. For females, fasting for one month did not impact cognition even after two weeks of fasting [76]. As we mentioned earlier, IF like KD, relies on carbohydrate deprivation to produce ketosis. Therefore, it is hard to speculate on the ketogenic effect if cognition is being tested after breaking a fast within the protocol.

Some studies showed that fasting may actually worsen cognition in healthy individuals [73,77,78]. Doniger et al. reported poorer cognitive performance across multiple cognitive domains, including memory, attention, motor, verbal and, executive functions in young men and women during fasting, especially later in the day as fasting progressed [78]. Other studies demonstrated initial [79] and persistent cognitive enhancements due to fasting [80].

Multiple studies examined glucose level changes with fasting regarding cognition [74,79,81,82]. Lower physiological glucose levels were detected in healthy individuals with fasting-related cognitive deficits; however, the association is undetermined [74,79,82]. Moreover, the consumption of glucose can reverse deteriorating cognition in generally healthy individuals [81]. In contrast, ketone bodies are under-reported in fasting studies, and it is hard to predict their role on cognition during fasting. IF ketosis requires long-term metabolic adaptations to appear [72]; most of the fasting studies performed on healthy and young individuals have not exceeded one month of fasting, and they mostly resemble Ramadan fasting [73,74,75,76,79]. Others tested fasting in a single-day setting [73,78,81], which could have been met with psychological difficulties that require adaptation and impact the study outcomes [83]. 

In a review by de Cabo and Mattson [84], which emphasized the favorable effect of IF on cognition, most of the included studies represented calorie-restricted protocols rather than IF, perhaps making the studies less comparable. The literature is heavily based on religious-fasting protocols [72,73,74,75,76,78,80]; thus, future researchers should be wary that religious fasting is not identical to the trending time-restricted fasting. Muslims are bound to abstain from any foods or drinks from dawn to sunset during the month of Ramadan, which varies between countries and is coupled with changes in sleep and circadian rhythms [80,85]. Typical trending time-restricted fasting (early or late) protocols include the night-time in fasting periods with a mid-day feeding window, and, unlike Ramadan fasting, permit non-caloric drinks (e.g., water).

Failure to demonstrate cognitive advantages in young and healthy individuals with full cognitive integrity [68,74,78] could be attributed to ceiling effects on initial cognitive performance or possible substrate metabolism efficiencies. In addition, IF effects could be more prominent in certain domains [73], which could be missed in many research designs.

## 5. Neuroimaging Assessment of Ketogenic Approaches and Cognitive Neurophysiology

### 5.1. Bioenergy Substrates and Brain Cognitive Function

As a highly active tissue, the brain requires use of a substantial amount of energy. At rest, the brain uses approximately 20% of the body’s O_2_ even though it only accounts for 2% of the body’s total weight [28]. Glucose is the primary energy source for all body tissues, and consequently, glucose metabolism is tightly regulated by hormonal factors and substrate availability [28]. However, brain glucose hypometabolism has been noted in AD, and ominous changes may occur earlier during MCI or as part of normal aging [86,87,88,89,90]. The findings of glucose hypometabolism were mostly drawn from positron emission tomography (PET) scans using fluorodeoxyglucose, but for translational research purposes, functional neuroimaging methods (e.g., fMRI, EEG, etc.) offer more-real life application to examine brain activation patterns upon glucose consumption and during cognitive workload. A few studies have demonstrated the impact of glucose on brain activation during cognitive workload and resting-state, most of which have noted increases in activation amplitude in response to higher induction of physiological glycemia [91].

As described in previous sections, ketones and IF could salvage cognitive functioning, especially in conditions where glucose hypometabolism could occur [86,87,88,89,90]. It is plausible to investigate how nutritionally induced ketosis affects the brain’s neurophysiology and whether it complements or surpasses glucose metabolism. Unlike glucose, ketone body uptake and metabolism seem to remain intact and efficient to provide the brain with its demand of nutrition, energy and neuroprotection [30,87,89]. Having cellular and molecular information on how ketosis affects the brain tissue is important [30,31], but it is necessary to expand research using functional neuroimaging methods to discover how the brain physiologically reacts to metabolic switching and how it could affect the brain during cognitively demanding activities. Very few and limited studies have been conducted within this context.

### 5.2. KD Ketosis Neurophysiological Implications for The Cognitive Brain

A randomized crossover trial by Neth et al. in older adults with pre-diabetes (N = 20) with cognitive changes and at risk for AD revealed that a ketogenic approach could provide cognitive benefits and prevention against cognitive decline [92]. Participants with subjective memory complaints (SMC) (*n* = 11) and MCI (*n* = 9) were assigned into two isocaloric 6-weeks interventions of a modified Mediterranean-ketogenic diet (MMKD) aimed at inducing ketosis or control low-fat American Heart Association Diet (AHAD) with a 6-week washout period in between (regular diet). Ketones were measured by capillary blood analysis, and it was found that MMKD markedly raised fasting ketones in the SMC and MCI groups (*M* = 1.0 and 0.4 mmol/L, respectively), which was met with a decrease in fasting blood glucose [92]. However, changes in blood ketones and glucose from baseline were not noted during AHAD. Upon fMRI data analysis, an increase in perfusion was detected in the left parahippocampal area and right temporal lobe in response to MMKD but not AHAD. When cognitive groups were compared, the MCI group displayed a perfusion increase in multiple brain regions, including the left medial and superior frontal regions, and the left inferior parietal and bilateral medial temporal regions. Lastly, no specific regions of greater perfusion were identified in the SMC group compared to MCI [92].

Moreover, it was shown that MMKD facilitated cerebral uptake of ketones while glucose uptake remained unaffected as measured by a novel dual-tracer PET scan [92]. This finding was similar to what was demonstrated by Castellano et al. [89], who showed that glucose metabolism could be affected by age while ketone metabolism remain efficient even after 4-years of follow up. No significant changes in uptake of either ketones or glucose were detected in AHAD, which highlighted the concept that brain ketone uptake may be related to the peripheral availability of ketones [93,94]. Regarding cognitive tests, MMKD and AHAD led to improvements in the Free and Cued Selective Reminding Test in both SMC and MCI (*p* ≤ 0.05) [92]. There was no improvement on other memory related tests (ADAS-Cog and Story Recall), however, and the authors alluded to the factor of a practice effect in the Free and Cued Selective Reminding Test [92]. In addition, this study demonstrated that when ketones are abundant in the blood, they can improve cerebral blood perfusion which facilitates their cerebral uptake regardless of glucose levels [92]. The findings align with a prior MCT-based study by Fortier et al. [25] that showed a global increase in cerebral metabolic rate (CMR) with ketone bodies in MCI participants without the influence of glucose. It is important to examine how this specific domain interacts with the state of ketosis by performing the cognitive tests while connected to a functional neuroimaging tool.

### 5.3. Ketosis Acute Effect on Neurocognition

We mentioned in a previous section that an MCT-based ketogenic meal improved cognition in healthy older adults [49]. The acute ketogenic energy supply to the brain generated by the swift MCT metabolism could be partly responsible for this outcome. fMRI data have shown unique neurophysiological patterns during cognitive workload and after the consumption of the same MCT formula in a similar set of participants [95]. In correspondence to an improvement in inhibitory control performance after 90 min of MCT consumption, blood-oxygenation-level-dependent (BOLD) signals were reduced at the right dorsolateral PFC in participants with initial lower global cognitive function (measured by MMSE) [95]. In contrast, participants with higher global cognitive function and improved working memory after MCT consumption showed a reduced increase in BOLD signals at the bilateral dorsolateral PFC when conducting a related test (i.e., N-Back) [95]. BOLD signals in fMRI and functional near-infrared spectroscopy (fNIRS) represent changes in oxygenated and deoxygenated hemoglobin levels in response to evoked neuronal activation through changes in neurovascular and neurometabolic coupling [96,97].

The ketosis induced by MCT consumption in Yomogida et al. research [95] clearly had an influential effect on the cognitive brain physiology and biochemistry; ketones sufficiently met the need of the demanding brain during cognitive workload that led to improvement in cognitive functioning. It is intriguing that the activation amplitude finding is somewhat comparable to that for glucose when it is supplemented to the demanding cognitive brain in younger individuals [98]. This highlights the notion of supporting brain bioenergy to enhance cognition as a concept requiring more in-depth evaluation and exploration. The presence of a discrepancy between groups based on global cognitive function [95] has an important clinical implication reflecting the possibility that ketogenic interventions are not one size that fits all but rather interventions that needs to be tailored individually. Future research efforts should aim to provide more insight into precision nutrition with KD as a marker of cognitive function in AD.

### 5.4. Fasting Neurophysiological Outcomes: Bioenergy Switch

With IF, an fMRI study by Chechko et al. showed certain cortical manifestations related to a 14-h overnight fast and blood glucose concentration during a working memory test [99]. Forty equally distributed young females and males with BMI categorized as within the recommended range were randomly assigned to receive glucagon and NaCl over two different sessions prior to fMRI scanning and after 14-h of overnight fasting. Blood glucose concentrations were physiologically lower than 80 mg/dL after fasting in both conditions and remained low after NaCl administration; however, glucose concentration gradually increased and peaked after 30 min of glucagon administration [99]. In the presence of low blood glucose levels, fMRI data revealed reduced recruitment of the bilateral rostral PFC, thalamus, basal ganglia, and posterior cingulate cortex during engagement in N-back tasks [99]. Based on these data, the researchers concluded that the bilateral midline thalamus and basal ganglia are most sensitive to blood glucose level changes, and an overnight fast for 14-hours can impact brain activation but not performance during memory-related tests. Again, this study demonstrated the brain’s sensitivity to metabolic changes but did not examine ketone bodies after fasting, especially because the last blood glucose test (at 15-h of fasting), was below even 70 mg/dL, which could have been met with an increment in ketone bodies. Knowing that ketones are the brain’s alternative fuel to glucose, it begs the question of whether ketones affect the same regions of the brain as glucose and whether ketones initiate an alternative compensatory activation pathway. Further research is needed to answer such queries for the field.

With fNIRS, 48-h of prolonged fasting in healthy young males showed a decrease in resting brain oxygenated hemoglobin (*p* = 0.040, *ES* = 0.64) without changes in deoxygenated and total hemoglobin on the dorsal and inferior PFC [100]. In this same study, glucose decreased from an average of 4.81 (*sd* = 0.65) to 3.74 (*sd* = 0.60) mmol/L after the fasting period, and the correlation between oxygenated hemoglobin and glucose was positively significant (*r* = 0.55, *p* = 0.47) [100]. Cognitive tests from this experiment indicated that only reaction time on the Two-Choice Reaction Time test (TCRTT) and Switching Task (ST) were significantly decreased. This demonstrates an improved response to fasting. With regard to fNIRS data during cognitive testing, TCRTT performance accuracy had the tendency of being negatively correlated with oxygenated hemoglobin (*r* = −0.5, *p* = 0.07), but the reaction time on the ST and mathematical processing task had a significant positive correlation with oxygenated hemoglobin [100]. In this study, ketones were not measured; however, they are expected to rise after 48 h of absolute fasting with declining glucose concentrations. The improvement observed in some cognitive tests and the fNIRS changes (during resting and cognitive tasks) could have been influenced by the ketone bodies instead of glucose given that peripheral ketosis from prolonged fasting govern the uptake of ketones in the brain with a significant correlation found (r = 0.86, *p* < 0.0001) [93]. Improvements in switching tasks with fasting was unlike what was found by Gagnon et al. [101], where ingestion of 50 g of glucose led to better dual-task coordination in conjunction with increased prefrontal region activation, as measured also by fNIRS. This finding further highlights the element of metabolic switching and substrate availability and the need to measure both ketones and glucose when a fasting protocol is administered.

### 5.5. ApoE4 Carriers: Neurophysiological Explanation

We have described the possible resistance of ApoE4 carriers to ketosis-related cognitive improvements in previous sections of our review. That was partially explained by a PET study that showed an increase in regional cerebral blood flow (rCBF) in ApoE4 negative individuals with mild to moderate AD after 45 days of daily 40 g ingestion of a ketogenic MCT (caprylidene) [102], whereas ApoE4 positive participants did not demonstrate such effects. Additionally, increased activation at the superior temporal gyrus, bilateral anterior cerebellum, right hypothalamus, and left inferior temporal were noted in ApoE4− only [102]. Moreover, within only 90-min, the first consumption of MCT was responsible for a greater activation at multiple brain areas in ApoE4− participants compared to ApoE4+. Greater activation in these brain regions were noted acutely at the dorsolateral PFC and temporal cortex [102], presenting significant challenges associated with ApoE4 carriers and, in turn, AD treatment using a ketosis-driven bioenergy supply.

## 6. Discussion

When alarming data predict an overwhelming increase in AD cases [2], scientific research efforts to ease the disease progress are crucial; this concept is apparent for any disease and was most recently witnessed during the COVID-19 pandemic. Lately, research examining the effects of various dietary manipulations to treat multiple neurocognitive diseases is advancing to build solid evidence for ultimate clinical translation. Because ketone bodies have been biochemically identified to be a substrate of bioenergetic relevance, and because initial research clues were suggestive of their ability to salvage failing glycolytic metabolism in AD affected brains [90,103,104], research using ketogenic approaches is justified.

This review focused on describing ketogenic interventions in relation to cognitive deterioration due to AD and its milder form (MCI) and contrasted research from healthy individuals. As a major organ, the brain requires a constant supply of energy to support its wide range of functions, including cognitive functions. The brain is able to uptake and actively metabolize ketone bodies once ketone bodies rise above the normal physiological level in the blood, regardless of glucose concentrations [93,94]. When glucose is underused, due to aging or disease [90], ketone bodies’ roles as an energy source and mitochondrial function support become very valuable, even in advanced AD cases [103].

Due to KD’s dietary intake structure, the minimal intake of carbohydrates and higher intake of fats drive the liver to produce ketone bodies to a higher physiological edge (0.5–3 mM) than that seen during a regular diet (<0.3 mM) [26]. Although some studies we mentioned found coupled cognitive advantages, the superiority of KD over other calorically restrictive protocols is still undetermined. With ketosis, ketone bodies may prohibit AD progression via anti-inflammatory processes known to influence disease pathology [24]; however, weight loss can trigger anti-inflammatory processes [105] as well, and is a common outcome shared between KD and other calorically restrictive regimens.

MCT’s ketogenic effect on the cognitive brain seems to be reproduced in multiple studies. Products that promote nutritional ketosis (i.e., MCT) could be superior to KD in terms of long-term compliance, especially in those with cognitive impairment who may need to maintain a steady state of ketosis [106]. However, when MCT is not combined with a diet such as KD, it can only drive ketogenesis for a few hours. The existing evidence of using MCT supplementation to support cognitive functioning in those experiencing cognitive decline is promising [43,44]. As described, it suggests that the beneficial effect of supplemental MCT on cognition is a consequence of a physiological shift to ketonemia [43,45,46,49], but continuous maintenance could be required to retain the cognitive benefits [44]. Cognitive enhancements are not limited to those with altered cognition; MCT supplements may advance cognitive performance in young individuals [47] and older adults free from dementia [49]. Multiple research protocols broke down total MCT consumption throughout the day, which probably created bouts of ketosis that might have more pronounced effects than a single dose. Research results are variable regarding dosage, formulas (C8 vs. C10), and mixtures. For clinical relevance, the minimum required doses in different groups with appropriate mixtures that ease side-effects should be identified and studied more extensively.

IF and KD share the same element of carbohydrate depletion to turn on hepatic ketogenesis [26,28], but it is worth highlighting that the amount of ketone bodies produced by the current popular IF regimens would probably not exceed the amount produced by MCT or KD. Human studies on the effect of IF on AD and neurocognitive diseases are still in their infancy [68]. Hence, the currently available data provide only preliminary clues about IF and cognitive preservation. It is possible that IF ketosis creates advantageous cognitive outcomes that are limited to older cognitively impaired adults [72]. However, future research should strengthen the body of evidence by (1) examining trending IF protocols instead of only religious-based practices; (2) permitting longstanding protocols as IF is deemed to be safe and requires practice adaptation [83,107]; and, (3) embedding neurophysiological investigation together with metabolic analyses to determine the ketogenic influence. Lastly, because IF is becoming a modern weight-loss method, research about long-term safety, metabolic changes, and cognitive consequences is needed for all age groups, regardless of initial cognitive status.

Neuroimaging modalities have revealed some neurophysiological characteristics associated with ketosis in relation to cognition [25,92]. As opposed to resting states, cognitive workload induces neuronal activation that can be detected via functional neuroimaging techniques (e.g., fMRI); the activation area is primarily dependent on the cognitive test and its primary correlated domain location within the brain [3]. Given the active metabolism of ketones that feeds into the energy cycle [94], it is possible that ketogenic metabolic sufficiency aids the cognitive brain; that was demonstrated by the regional decrease in activation reported in some studies with ketosis probably reflecting metabolic sufficiency during cognitive workloads [95,100]. More focused studies are henceforth needed to confirm whether ketosis does, in fact, influence brain activation potentials that, in turn, assist with neurocognitive functions.

Ketogenic methods to rescue the brain from cognitive deterioration related to aging, MCI, and potentially AD, are possible but still indefinite. It is possible that cognition can only be improved in those with initial cognitive deficits, and that younger and cognitively intact persons may not experience cognitive enhancements with ketosis. Studies to date have focused mainly on metabolically healthy individuals; however, it is sensible to acquire a broader set of data from individuals with comorbidities and chronic metabolic diseases (e.g., DM) for two main reasons: (1) their association with cognitive deterioration [108]; and, (2) the presence of irregular metabolic status that can interfere with ketosis maintenance. 

Compared to pathological diabetic ketoacidosis (DKA), physiological ketosis can reach a limit of 8 mM without associated acidosis [109]. It is possible that individuals with Type 1 [110] and Type 2 DM [111,112] can safely adhere to a ketogenic lifestyle, but more data is needed in this regard. Concerning lipid profiling, some studies have reported that MCT interventions modestly raised blood lipids [43,44,46]. In recent systematic reviews and meta-analyses they were found to significantly increase triglycerides [113], and diets rich in MCFA compared to LCFA increased HDL [114]. However, these studies found no significant impact on LDL concentrations [113,114]. However, it is still undetermined whether high-fat intake associated with KD and MCT consumption can eventually lead to cardiovascular risk [115] and liver damage (non-alcoholic fatty liver disease; NAFLD) [116], and whether prolonged ketosis can lead to adverse events. Future studies must therefore determine the safety of prolonged interventions and weigh their benefits. Another ketogenic method was not described in our review, namely exogenous ketones (ketone ester and salts). We intended to describe methods that are more publicly disseminated.

The presence of the ApoE4 allele bears a major resistance issue to ketogenic interventions against cognitive deterioration in MCI and AD; however, it is still possible to obtain ketogenic cognitive benefits in heterozygous carriers when mixed with lifestyle modifications [38] and double ketogenic approaches [39]. The possible issue could be the inability of the brain to acquire and use ketone bodies despite their profuse availability [102]. It is crucial to intensify research to provide further neurophysiological and molecular explanations to overcome disease progress in ApoE4 carriers that pose a higher risk of AD [37].

Ketogenic protocols are unequal in regard to ketosis. KD and IF require constant adherence to establish ketosis as opposed to MCT, which can trigger ketosis in a matter of minutes [41]. Future research should determine the ideal feasible method and sub-method (e.g., time-restricted IF vs. alternate-day IF), or minimum-effective-dose of MCT and composition (i.e., C8 vs. C10) that can produce cognitive advantages. Novel methods, such as MCT-supplemented IF or KD-combined IF [39], are worth examining. Moreover, the identification of the minimum ketosis threshold that can produce favorable cognitive outcomes, regardless of method, is essential for clinical relevance. We wish to emphasize that molecular and neurophysiological explanations are highly valued at this point with respect to their research potential, and acknowledge that mechanistic research may have promise in bolstering the evidence within this context and is needed to advance ketogenic use to manage cognitive decrements.

## Figures and Tables

**Table 1:** Research trials that examined the effect of MCT-induced ketosis on cognition.

Reference	Intervention			Outcomes	
	Sample	Test	Duration	Ketones	Cognition
Taylor et al., 2017 [44]	N = 10, very mild to mild AD	MCT supplemented very high-fat KD	3 months	BHB increase from average Mean (M) = 0.11 to 0.31 mmol/L (*p* < 0.001)	4.1 points improvement on ADAS-Cog scale (*p* = 0.02)
Fortier et al., 2021 [43]	N = 122, MCI (Age ≥ 55)	15 g bi-daily MCT drink (*n* = 39) without dietary restriction	6 months	Total ketones increase after MCT for <4 h	Improvement on multiple cognitive domains post-intervention
Rebello et al., 2015 [45]	N = 4, MCI (Age ≥ 50)	56 g/day MCT oil (*n* = 2)	6 months	BHB increase postprandially	Cognitive improvement on ADAS-Cog
Xu et al., 2020 [46]	N = 49, mild to moderate AD (Age ≥ 55)	17.3 g/day MCT jellies with meals	1 month	BHB (*p* < 0.01) and acetoacetate (*p <* 0.01) increase	Improvement limited to those who were ApoE4− (*n* = 46) in multiple domains measured by ADAS-Cog
Ashton et al., 2020 [47]	N = 30, healthy participants (Mean age = 19.7. SD = 1.5)	18 g/day or 12 g/day MCT gels	1 month	Unmeasured	Improvement in cognition in both MCT doses
Ota et al., 2019 [48]	N = 20, Mild to moderate AD (Mean age = 73.4. SD = 6.0)	20 g MCT ketogenic meal	Single test day and 3 months	BHB and acetoacetate increase (*p* < 0.001) after consumption but not persistent	Improvements in memory and processing speed after 3 months only
Ota et al., 2016 [49]	N = 19, healthy undemented adults (Age > 60)	20 g MCT ketogenic meal	Single test day	BHB and acetoacetate increase (*p* < 0.001)	Instant improvement of cognitive functions
Reger et al., 2004 [50]	N = 20, Participants with probable AD or amnestic MCI (Mean age = 74.7. SD = 6.7)	40 mL MCT containing drink	Single test day	BHB increased above 0.5 mM after 2 h	Improvement limited to those who were ApoE4− (*n* = 10) as measured by ADAS-Cog
